# Food allergy masquerading as persistent proteinuria in post-infectious glomerulonephritis: a case report

**DOI:** 10.1093/omcr/omad087

**Published:** 2023-08-20

**Authors:** Masoumeh Mohkam, Mahnaz Jamee, Nafiseh Mortazavi, Mahbubeh Mirzaee, Mahboubeh Mansouri

**Affiliations:** Pediatric Nephrology Research Center, Research Institute for Children's Health, Shahid Beheshti University of Medical Sciences, Tehran, Iran; Pediatric Nephrology Research Center, Research Institute for Children's Health, Shahid Beheshti University of Medical Sciences, Tehran, Iran; Immunology and Allergy Department, Mofid Children's Hospital, Shahid Beheshti University of Medical Sciences, Tehran, Iran; Pediatric Pathology Department, Iran University of Medical Sciences, Tehran, Iran; Pediatric Nephrology Research Center, Research Institute for Children's Health, Shahid Beheshti University of Medical Sciences, Tehran, Iran; Immunology and Allergy Department, Mofid Children's Hospital, Shahid Beheshti University of Medical Sciences, Tehran, Iran

## Abstract

Background: Post-infectious glomerulonephritis (PIGN) is one of the most common causes of pediatric acute glomerulonephritis. Immune system dysregulation manifesting as food allergy may predispose PIGN patients to nephrotic-range proteinuria.

Case Presentation: The patient was a 3-year-old male that presented with edema, gross hematuria and reduced urine output following a mild fever, rhinorrhea and lethargy. Due to the persistence of proteinuria and hematuria, he underwent a kidney biopsy. The patient was diagnosed with atypical PIGN and was placed on oral prednisolone. During treatment, a relationship between the consumption of dairy products and the degree of proteinuria was noted. The clinical manifestations and urinalysis indices improved upon steroid discontinuation and initiation of a hypoallergic diet.

Conclusion: The association between the degree of proteinuria and consumption of dairy products in this PIGN patient led to the identification of food allergy as an underlying factor for nephrotic-range proteinuria.

## INTRODUCTION

Post-infectious glomerulonephritis (PIGN) is one of the most common causes of acute glomerulonephritis in children. Most cases of PIGN occur after a streptococcal infection [1].

Studies have shown that one episode of PIGN in childhood can predispose patients to an increased risk of chronic kidney disease [2]. Therefore, early diagnosis and initiating appropriate antimicrobial treatment for both treatment and prophylaxis are preventive steps to be undertaken [3].

The amount of proteinuria in PIGN may be related to the patient’s immune system response [4, 5]. In addition, the terminal blockade of the complement pathway via an anti-C5 monoclonal antibody (eculizumab) has been promising in PIGN [6].

This case report presents a 3-year-old boy with a new-onset PIGN who had persistent proteinuria under treatment with prednisolone. His proteinuria resolved after starting a hypo-allergen regimen. This case report may provide further evidence on the association between allergic disorders and PIGN.

## CASE PRESENTATION

The patient, a 3-year-old boy and the second child of Iranian non-consanguineous parents, presented with gross hematuria and edema. He had a history of lethargy, rhinorrhea and a mild fever 10 days before admission.

In the physical examination, the patient had normal growth (weight: 14.5 Kg (CDC percentile: 52%), height: 96 cm (CDC percentile: 59%)) and blood pressure (105/70 mmHg). Other physical examinations were normal. A summary of laboratory findings is presented in Table 1. In the laboratory evaluation, the urine volume was decreased to 1 cc/Kg/h. In the first urine analysis, many red blood cells (>60% dysmorphic red blood cell (RBCs)) and proteinuria (3+) were detected. The random urine protein/creatinine ratio was within the nephrotic range. The patient had anemia, hypoalbuminemia, hypertriglyceridemia and hypercholesterolemia. The serum creatinine level was high but gradually normalized in the next few days. The serum complement 3 level was initially low (C3: 75 mg/dL) but then normalized (C3: 88 mg/dL).

**Table 1:** Summary of laboratory findings in the first presentation

Parameter	Result	Normal range
Hemoglobin (gr/dL)	8	11.5–16.5
Random Urine Pro/Cr) mg/mg)	8.3	<0.2
Albumin (gr/dL)	2.1	3.4–5.4
Triglyceride (mg/dL)	175	<150
Total cholesterol (mg/dL)	220	<170
Serum Cr (mg/dL)	1.4	<0.5
C3 (mg/dL)	75	80–160

Pro; protein, Cr; creatinine, C3; complement 3.

**Figure 1:**
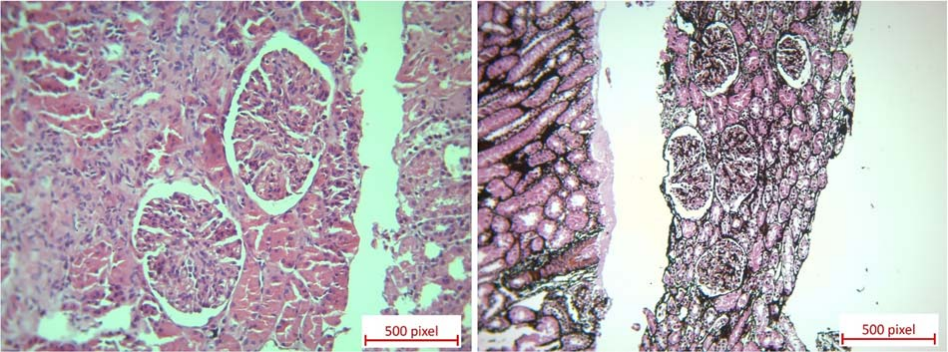
All glomeruli in the specimen show mesangial hypercellularity but a few of them have segmental endocapillary proliferation with polymorphonuclear cell infiltration. There are very rare foci of GBM thickening. These pathological findings are suggestive of the resolving stage of PIGN.

The primary diagnosis was PIGN. Due to persistent proteinuria and gross hematuria with variable severity, he underwent a kidney biopsy 6 weeks later. The results showed mesangial hypercellularity in all glomeruli, segmental endocapillary proliferation with polymorphonuclear cell infiltration and rare foci of glomerular basement membrane (GBM) thickening (Fig. 1). Immunofluorescence staining, scattered capillary and mesangial IgG and C3 deposits along GBM and mesangium. Based on these findings and the clinical course, a diagnosis of atypical PIGN was made.

Two months later, the UA showed mild proteinuria and 50–60 RBCs. The random urine protein/creatinine ratio was 6.0. The refractory form of PIGN was suspected and treatment with prednisolone 2 mg/kg was started. Within the first week of corticosteroid administration, the patient developed hypertension and epistaxis; therefore, captopril 1 mg/kg was added to the regimen. Two weeks later, hypoalbuminemia (Alb: 2.1 g/dL) following nephrotic-range proteinuria (U/A protein: +1 and urine protein 24 h: 1524 mg/dL) was detected. Five days later, proteinuria (U/A protein: +1 and urine protein 24 h: 770) and hematuria (U/A RBCs: 25–60) improved but persisted.

One month later, prednisolone was tapered to every other day. Captopril was later discontinued due to hypotension.

At the time, the serum C3 level was 183, serum albumin was 2.3 g/dL, and the random urine protein/creatinine ratio of 1.5.

The patient’s parents were medical nurses and evaluated him carefully using urine dipsticks every day. They noticed a relationship between the consumption of dairy products and the degree of proteinuria. When the patient received dairy products, the degree of proteinuria in the dipstick test increased from 1+ to 2+. Allergy consultation recommended food allergy-induced protein loss. Based on the prick test, the patient received a hypoallergic diet and prednisolone was discontinued. The patient’s anemia improved (Hb: 11 mg/dL), serum creatinine decreased (Cr: 0.4 mg/dL), and serum albumin increased (Alb: 2.8 g/dL). Two weeks later, the urine dysmorphic RBCs reduced to 15% and the 24 h protein level decreased to 198 mg/dL) (protein/Cr ratio: 0.8). Three weeks after commencing the hypoallergic regimen, the U/A showed 1+ proteinuria and 5% dysmorphic RBCs. Three weeks later, no further proteinuria was evident. In the last follow-up 1 year after starting a hypoallergic diet, the patient had no other manifestations, and the U/A was completely normal.

## DISCUSSION

In our patient, the association between the amount of proteinuria and consumption of dairy products led to the identification of food allergy as an underlying factor for nephrotic-range proteinuria and steroid resistance.

The parents noticed his proteinuria increased after eating dairy products. The patient responded well to diet modification. It was hypothesized that the cause of proteinuria could be food allergy. We believe the patient’s allergy was non-IgE mediated or a mixed-type allergy. In this type of allergy, laboratory tests cannot confirm the disease. To confirm the disease, the patient should follow a diet and avoid allergens for at least 6 months. If the patient’s symptoms do not recur, the diagnosis is confirmed. A negative history of allergy in the patients or their family members does not rule out the probability of its occurrence.

The relationship between allergic disorders and increased serum IgE levels and glomerulopathies such as nephrotic syndrome is well established [7]. Yet, the impact of allergy on the persistence of proteinuria in PIGN is not reported. In 1987, J. Laurent et al. evaluated the role of food antigens in idiopathic nephrotic syndromes. The result of this study was as follows, that food can be responsible for idiopathic nephrotic syndrome [8]. In a population-based cohort among children, the incidence of nephrotic syndrome was 3.36-fold higher in those with asthma compared to children without it [9].

Previous studies have introduced interleukin 13 as a stimulator of the IgE response that may be involved in the pathogenesis of proteinuria in patients with minimal change disease [7]. In 2020, Koochaki *et al.* evaluated the role of a specific diet on the amelioration of asymptomatic microscopic hematuria in 122 children and found a significant decrease in the number of RBCs in UA [10].

## CONCLUSION

As observed in this patient, the food allergy can trigger persistent proteinuria in certain conditions such as PIGN. However, these observations warrant more research.

## References

[1] Nasr SH, Radhakrishnan J, D'Agati VD. Bacterial infection-related glomerulonephritis in adults. Kidney Int 2013;83:792–803. 23302723 10.1038/ki.2012.407

[2] Oda T, Yoshizawa N. Factors affecting the progression of infection-related glomerulonephritis to chronic kidney disease. Int J Mol Sci 2021;22. 10.3390/ijms22020905 PMC7831296 33477598

[3] Alhamoud MA, Salloot IZ, Mohiuddin SS, AlHarbi TM, Batouq F, Alfrayyan NY et al. A comprehensive review study on glomerulonephritis associated with post-streptococcal infection. Cureus 2021;13:e20212. 35004032 10.7759/cureus.20212 PMC8730744

[4] Zhou C, Liu Z, Sui W, Gu D, Li Y, Zou H. [detection of serum food specific antibodies of 6 common foods in patients with IgA nephropathy]. Nan fang yi ke da xue xue bao =. J Southern Med Univ 2014;34:419–22. 24670462

[5] Kloster Smerud H, Fellström B, Hällgren R, Osagie S, Venge P, Kristjánsson G. Gastrointestinal sensitivity to soy and milk proteins in patients with IgA nephropathy. Clin Nephrol 2010;74:364–71. 20979945 10.5414/cnp74364

[6] Jamee M, Farsi Y, Akhondi AH, Kamali F, Hajialigol AH, Mirzaee M et al. Eculizumab in Pediatric kidney disorders: a review: Eculizumab in kidney disorders. J Pediatric Nephrol 2022;10:103–11.

[7] Abdel-Hafez M, Shimada M, Lee PY, Johnson RJ, Garin EH. Idiopathic nephrotic syndrome and atopy: is there a common link? Am J kidney Dis Off J Nat Kidney Foundation 2009;54:945–53. 10.1053/j.ajkd.2009.03.019 PMC2895907 19556042

[8] Laurent J, Rostoker G, Robeva R, Bruneau C, Lagrue G. Is adult idiopathic nephrotic syndrome food allergy? Value Oligoantigenic Diets Nephron 1987;47:7–11. 3627337 10.1159/000184448

[9] Wei CC, Lin CL, Shen TC, Li YF. Risk of idiopathic nephrotic syndrome among children with asthma: a nationwide, population-based cohort study. Pediatr Res 2015;78:212–7. 25927542 10.1038/pr.2015.80

[10] Koochaki R, Mohkam M. The role of specific diet on the treatment of Pediatric asymptomatic microscopic Hematuria; a before and after study: specific diet on microscopic Hematuria. J Pediatric Nephrol 2020;8:1–4.

